# *Juniperus communis* extract ameliorates lipopolysaccharide‐induced acute kidney injury through the adenosine monophosphate–activated protein kinase pathway

**DOI:** 10.1002/fsn3.2941

**Published:** 2022-07-02

**Authors:** Ta‐Chin Lin, Chia‐Wen Lu, Kai‐Fu Chang, Chung‐Jen Lee

**Affiliations:** ^1^ Department of Surgery, National Defense Medical Center Tri‐Service General Hospital Penghu Branch Magong City Taiwan; ^2^ Department of Nursing Buddhist Tzu Chi General Hospital Hualien Taiwan; ^3^ Department of Medical Laboratory and Biotechnology Chung Shan Medical University Taichung Taiwan; ^4^ Department of Nursing Tzu Chi University of Science and Technology Hualien Taiwan

**Keywords:** acute kidney injury, adenosine monophosphate–activated protein kinase, *Juniperus Communis*, nuclear factor‐κB

## Abstract

Septic shock can aggravate organ dysfunction and even lead to death. *Juniperus communis* (JCo) extract has been experimentally demonstrated to have anti‐inflammatory and antioxidant effects. We investigated the anti‐inflammatory and antioxidant mechanism of JCo extract in vivo and in vitro. In a lipopolysaccharide (LPS)‐induced acute kidney injury rat model, JCo extract improved animal survival, reduced kidney injury scores, suppressed kidney injury molecule‐1, and preserved E‐cadherin expression from LPS damage, as demonstrated by the immunohistochemistry examinations of the rat kidneys. In LPS‐stimulated NRK‐52E cells, JCo extract inhibited nuclear factor‐κB (NF‐κB) and increased adenosine monophosphate–activated protein kinase (AMPK) expression, prompting the activation of the antioxidant nuclear factor erythroid 2–related factor‐2/heme oxygenase‐1 pathway against oxidative stress. JCo extract ameliorated LPS‐induced acute kidney injury by suppressing NF‐κB signaling and stimulating the release of tumor necrosis factor‐α and interleukin‐1β through the AMPK pathway.

## Graphical abstract

JCo extract ameliorated LPS‐induced acute kidney injury by suppressing NFκB signaling and releasing tumor necrosis factor‐α and interleukin‐1β through the AMPK pathway.

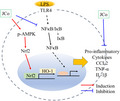

## 1 INTRODUCTION

Sepsis refers to life‐threatening organ dysfunction associated with infection. It is a leading cause of acute kidney injury (AKI) in patients with critical illness, and 60% of patients with sepsis present with AKI (Poston & Koyner, 2019; Skube et al., 2018). Injected lipopolysaccharide (LPS), a major component of the outer membrane of gram‐negative bacteria, can induce sepsis and mimic the infection observed in humans (Kupferschmid et al., 2018). Following LPS injection, renal tubular epithelial cells express toll‐like receptor 4 (TLR4), and the binding of the LPS to TLR4 activates the downstream signaling pathways, including the nuclear factor‐κB (NF‐κB) pathway; this activation triggers an inflammatory response involving increased synthesis of proinflammatory cytokines and reactive oxygen species (ROS) as well as oxidative stress, ultimately leading to multiple‐organ dysfunction (Liu & Malik, 2006; (Peerapornratana et al., 2019)). However, adenosine monophosphate–activated protein kinase (AMPK) can suppress inflammation, inhibit NF‐κB signaling (Salt & Palmer, 2012), and promote the activation of the antioxidant nuclear factor erythroid 2–related factor 2 (Nrf2)/heme oxygenase (HO)‐1 pathway against oxidative stress (Lei et al., 2020; Zimmermann et al., 2015).

Natural products, including traditional and herbal medicines, have been applied in the development of medical applications. *Juniperus communis* (JCo) is an evergreen dioecious shrub of genus *Juniperus* of the cypress family Cupressaceae (Hajdari et al., 2015). JCo extract contains α‐pinene, β‐pinene, apigenin, sabinene, β‐sitosterol, campesterol, limonene, and cupressuflavone, among other compounds (Bais et al., 2014; Li et al., 2021). JCo extract has been experimentally demonstrated to have antioxidant, antibacterial, antiviral, antifungal, anticancer, and anti‐inflammatory effects (Huang et al., 2021; Moein et al., 2010; Raina et al., 2019), thus making it a potential therapeutic agent. However, few studies have investigated the anti‐inflammatory and antioxidant mechanism and pathophysiology of JCo extract in vitro and in vivo.

We explored the potential effects of JCo extract on AKI in rats with LPS‐induced endotoxic shock and in LPS‐stimulated NRK‐52E cells. Our results indicated a restorative effect of JCo extract on the histopathological changes related to LPS‐induced kidney injury through the reduction in proinflammatory cytokine production through the AMPK pathway.

## 2 MATERIALS AND METHODS

### 2.1 Preparation of animals

A total of 32 male Sprague–Dawley rats weighing between 310 and 330 g (10–11 weeks old) purchased from BioLASCO were used in experiments. All animals were maintained in the Laboratory Animal Center of Tzu Chi University (Hualien, Taiwan). The research protocol for animal use was approved by the Institutional Animal Care and Use Committee of Tzu Chi University of Science and Technology.

### 2.2 Vascular catheterization

For blood pressure analysis, rats were anesthetized using isoflurane (Baxter Healthcare) inhalation (Matrx VIP 3000). Subsequently, the femoral artery and femoral vein were cannulated with PE‐50. The procedure was completed in 15 min, leaving a small wound (<0.5 cm^2^); thereafter, each rat was placed in a metabolic cage (Lee et al., 2009). The femoral artery was connected to a pressure transducer to record arterial pressure (E‐corder 410, eDAQ, Australia). Endotoxic shock was induced 12 h later with the rats in a conscious state. The femoral vein was catheterized for intravenous administration of drugs or fluid.

### 2.3 Rat model of endotoxic shock

Endotoxic shock was induced through injection of LPS (*Escherichia coli* O111:B4; L2630, Sigma‐Aldrich) at a dose of 20 mg/kg body weight (dissolved in 1 ml of 0.9% normal saline) administered for 30 min.

### 2.4 Experimental design

**FIGURE 1:**
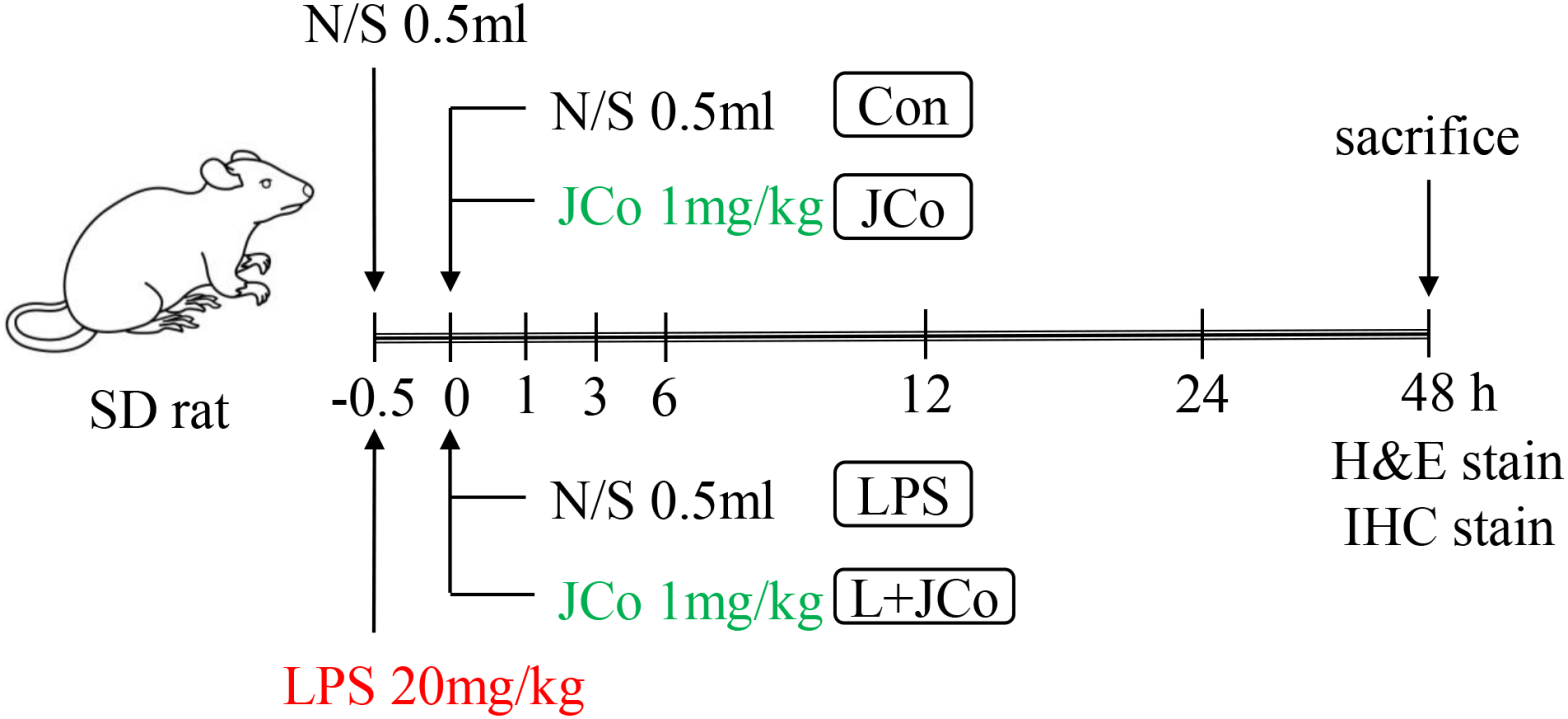
Experimental design of the animal study. LPS (20 mg/kg body weight) in 0.9% normal saline was injected through the femoral artery for 30 min, with the end of LPS induction set as time 0. *N* = 8 in each group. L + JCo and LPS + JCo group

A total of 32 rats were randomly divided into four groups. The control group received 2 ml of normal saline (2 ml/h). The JCo group received JCo extract (1 mg/kg body weight in 1 ml of 0.9% normal saline) for 30 min, followed by 1 ml of normal saline. The LPS and LPS + JCo groups received normal saline and JCo, respectively, for 30 min after LPS administration (Figure 1). All rats were observed for 48 h after LPS administration and then sacrificed as the end point.

### 2.5 Blood sample analysis

Blood samples (0.5 ml) were collected for the measurement of blood urea nitrogen (BUN) and creatinine (Cre) 1 h before the administration of LPS and 1, 3, 6, 9, 12, and 24 h after LPS injection. Blood samples were centrifuged immediately at 6708 *g* for 10 min. The serum was collected for biochemical analysis of BUN and Cre using a Spotchem SP‐4430 analyzer (Arkray) (Tsai et al., 2017). The serum levels of tumor necrosis factor‐α (TNF‐α) and interleukin‐1β (IL‐1β) were measured using commercial enzyme‐linked immunosorbent assay kits (Abcam).

### 2.6 Histological examination

Carbon dioxide was used to euthanize all animals. The collected kidneys were fixed in 10% formalin, embedded, and stained through hematoxylin and immunohistochemical staining. The severity of renal tubular injury was determined by estimating the percentage of tubules in the cortex or the outer medulla, demonstrating epithelial necrosis or the presence of luminal necrotic debris and tubular dilation, and was categorized as follows: 0, none; 1, <5%; 2, 5%–25%; 3, 25%–75%; and 4, >75% (Lee et al., 2009).

Sections of the kidneys were deparaffinized, rehydrated, and subjected to microwave‐assisted antigen retrieval (Trilogy, Cell Marque). Subsequently, the sections were subjected to endogenous peroxidase activity blocking with a 3% hydrogen peroxide solution for 5 min and 10% bovine serum albumin–containing phosphate buffered saline for 1 h at room temperature. Kidney injury molecule‐1 (KIM‐1) (AB47635 1:200 dilution, Abcam) and E‐cadherin (AB76055 1:200 dilution, Abcam) were used as primary antibodies. After three washes, the sections were incubated with biotinylated goat anti‐mouse secondary antibodies at room temperature for 30 min. The reaction was visualized with 3,3′‐diaminobenzidine and then counterstained with Mayer's hematoxylin, dehydrated with ethanol, and coverslipped for evaluation. The slides were observed under light microscopy, and immunohistochemical analysis was performed on the basis of the average optical density of positive reactions by using Image‐Pro Plus 6.0 software (Wu et al., 2021).

### 2.7 Cell culture and reagents

The normal NRK‐52E rat proximal tubular cells, obtained from the Food Industry Research and Development Institute (Hsinchu, Taiwan), were cultured in Dulbecco's modified Eagle medium (Sigma‐Aldrich) supplemented with 5% fetal bovine serum (Life Technologies), 4‐(2‐hydroxyethyl)‐1‐piperazineethanesulfonic acid (Sigma‐Aldrich), and penicillin–streptomycin (100 U/ml penicillin and 100 μg/ml streptomycin; Sigma‐Aldrich) in an incubator with 5% CO_2_ at 37°C.

The major components of the JCo extract used in this study were α‐pinene (34.87%), citronellyl acetate (14.26%), limonene (10.72%), trpinolene (10.65%), and p‐cymene (6.21%), as revealed using gas chromatography–mass spectrometry (Gao et al., 2019). *Juniperus communis* plant extract was obtained from Phoenix (Red Bank, ); extraction was conducted with reference to the method of using steam distillation (Lee et al., 2020). JCo extract and LPS were freshly dissolved in dimethyl sulfoxide (DMSO) and phosphate buffered saline, respectively. The final concentration of DMSO in the medium was less than 1%.

### 2.8 Semiquantitative reverse transcription‐polymerase chain reaction

In the experiments, four groups were employed: (1) control, (2) LPS, (3) JCo, and (4) LPS + JCo. NRK‐52E groups had their cells seeded at a density of 5 × 10^5^ cells on a six‐well culture plate and incubated overnight. The culture medium was replaced with serum‐free medium containing JCo (10 μg/ml) with or without LPS (10 μg/ml). After incubation for 12 or 24 h, RNA was extracted using TRIzol RNA Isolation Reagents (Genepure) in accordance with the manufacturer's instructions, and RNA concentration was determined through spectrophotometry. RNA was reverse transcribed to cDNA in the presence of primers from the HiSpec Reverse Transcriptase kit (Yeastern Biotech). A total of 40 polymerase chain reaction (PCR) cycles were performed using 5× Taq PCR MasterMix (Biomate) under the following conditions: 95°C for 30 s, 55–56°C for 30 s, and 72°C for 30 s. The primers used for PCR amplification are presented in Table S1 and include NF‐κB, chemokine ligand 2 (CCL2), IL‐1β, IL‐6, AMPK, Nrf‐2, and HO‐1. Consistent with the approach of Wu et al. (2021), PCR products were lysed using 2% agarose gel electrophoresis, stained with ethidium bromide, and photographed using the ProteinSimple Alphaimager HP System.

### 2.9 Western blotting

NRK‐52E cells (5 × 10^6^ cells) were seeded in a 10 cm culture dish overnight and treated with JCo (10 μg/ml) with or without 10 μg/ml LPS in serum‐free medium for 12 and 24 h, respectively, after which they were lysed in lysis buffer containing protease inhibitors (BioBasic). Lysates were then centrifuged at 12,000 *g* for 30 min at 4°C, and the protein content of the supernatant was calculated using a bicinchoninic acid protein assay kit (Pierce Biotechnology). Each protein sample (20 g) was separated using 8%–12.5% sodium dodecyl sulfate–polyacrylamide gel electrophoresis and transferred onto polyvinylidene fluoride membranes (FluoroTrans, PALL). The membranes were blocked with 5% nonfat dry milk and incubated overnight at 4°C with the following primary antibodies: NFκB p65, p‐NFκB p65, AMPK, and HO‐1 (1/200 dilution) and p‐AMPK, Nrf2, and p‐Nrf2 (1/1000 dilution; Novus Biologicals). The membranes were washed thrice with 0.5% Tween‐20 in TBS and incubated with biotin conjugated secondary antibodies (1/1500 dilution; Santa Cruz) for 2 h at room temperature and then with horseradish peroxidase–conjugated streptavidin for 1 h. Antibody‐reactive proteins were allowed to interact through enhanced chemiluminescence (T‐Pro Biotechnology) and were detected using a chemiluminescence–fluorescence imaging analyzer (GE LAS‐4000, GE Healthcare Life Sciences). The band of each sample was quantified using ImageJ 1.47 t software, and all results were normalized to the value of the control (Tsai et al., 2018).

### 2.10 Detection of ROS

The NRK‐52E cells were subcultured in a 12‐well plate at a density of 4 × 10^5^ cells and treated separately with 10 μg/ml LPS, 100 μM H_2_O_2_, 10 μg/ml JCo, and LPS + JCo extract in serum‐free medium for 12 and 24 h. After cell collection, reactive oxygen species (ROS) generation was assessed using 2′,7′‐dichlorodihydrofluorescein diacetate (DCF‐DA) staining in accordance with the manufacturer's instructions. After a wash with phosphate‐buffered saline, the cells stained with DCF‐DA and 4′,6‐diamidino‐2‐phenylindole were examined under fluorescence microscopy (Wu et al., 2021).

### 2.11 Statistical analysis

Data are expressed as the mean ± standard error of the mean. For multiple comparisons, significance was determined using a one‐way analysis of variance with Bonferroni's post hoc test (SPSS 19.0; SPSS, Inc.). An unpaired *t* test was used to identify significant differences between the two groups. The Kaplan–Meier method was used to estimate survival rates, and the log rank test was used to compare the mortality rates among the groups. Statistical significance was indicated by *p* < .05.

## 3 RESULTS

### 3.1 JCo extract attenuated endotoxic shock–induced renal damage, reduced inflammatory biomarker levels, and increased survival

**FIGURE 2:**
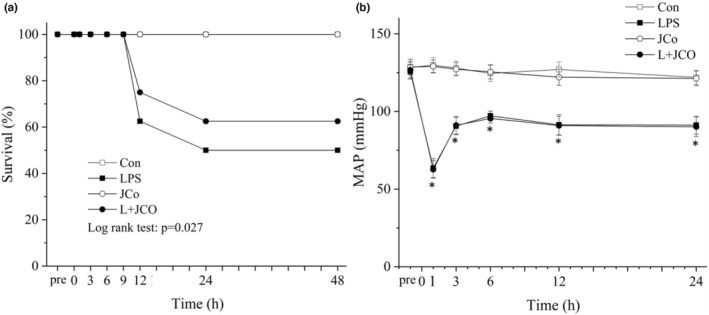
Changes in the (a) Kaplan–Meier survival curve and (b) MAP after LPS‐induced endotoxic shock in rats. *p < .05 for the LPS group compared with the control group. ^#^ *p* < .05 for the LPS + JCo group compared with the LPS group. Control (*n* = 8), JCo (*n* = 8), LPS (*n* = 4), and LPS + JCo (*n* = 5) groups at 48 h after LPS administration. L + JCo and LPS + JCo group

At 48 h after the induction of endotoxic shock with LPS, the survival rate was 50% in the LPS group, 62.5% in the LPS + JCo group, and 100% in the control and JCo groups (Figure 2A). All rats were alive until 10 h after LPS administration; however, at 12 h after LPS administration, three rats had died in the LPS group versus two deaths in the LPS + JCo group. The mortality rate in the LPS + JCo group was significantly lower than that in the LPS group (log rank test; *p* = .027).

The mean arterial pressure (MAP) rapidly decreased during the first hour after LPS administration. Blood pressure in the LPS and LPS + JCo groups was lower than that in the control and JCo groups in the 24 h after induction of endotoxic shock (Figure 2B). Posttreatment improvement in MAP did not differ significantly between the LPS + JCo and LPS groups.

**FIGURE 3:**
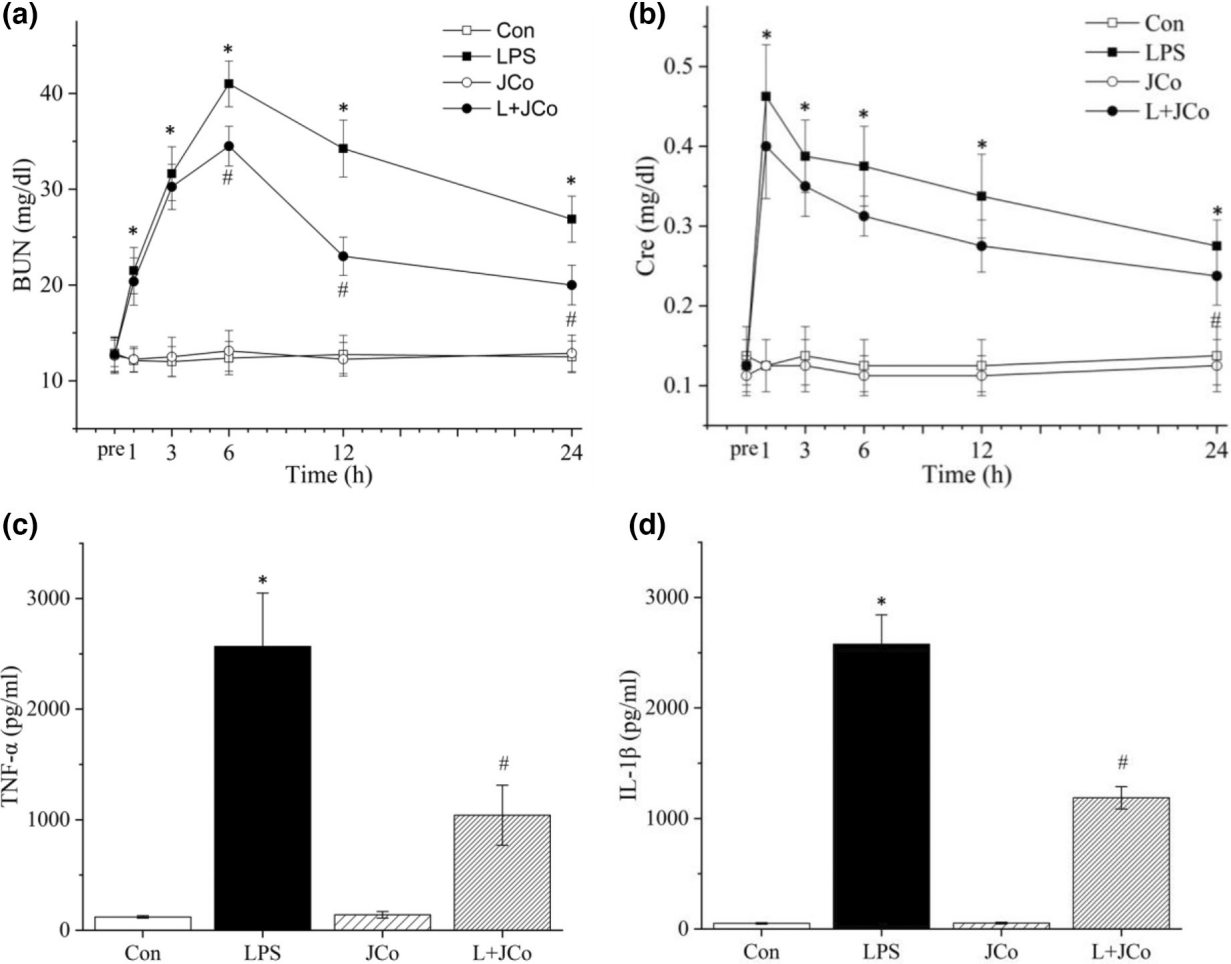
Levels of (a) BUN, (b) Cre, (c) TNF‐α, and (d) IL‐1β. LPS administration was completed at time 0. TNF‐α (*n* = 8 in each of the four groups) was assessed at 1 h, and IL‐1β was assessed at 3 h. *p < .05 for the LPS group compared with the control group. ^#^ *p* < .05 for the LPS + JCo group compared with the LPS group. L + JCo and LPS + JCo group

Peak BUN and Cre levels were observed at 6 and 1 h after endotoxic shock induction, respectively. Relative to the LPS group, the LPS + JCo group exhibited lower BUN levels at 6, 12, and 24 h after LPS administration (*p* < .05; Figure 3A). Increased serum Cre levels were observed 1 h after induction of endotoxic shock and remained elevated from baseline. Compared with the LPS group, the LPS + JCo group had lower Cre levels at 6, 12, and 24 h after LPS administration (*p* < .05; Figure 3B).

We measured the levels of serum inflammatory biomarkers TNF‐α and IL‐1β at 1 and 3 h after LPS administration, respectively (Figure 3). TNF‐α and IL‐1β levels increased following LPS administration; however, following JCo treatment, the LPS + JCo group had significantly lower serum TNF‐α (*p* < .05; Figure 3C) and IL‐1β levels (*p* < .05; Figure 3D) than did the LPS group.

### 3.2 JCo extract attenuated endotoxic shock–induced renal damage

**FIGURE 4:**
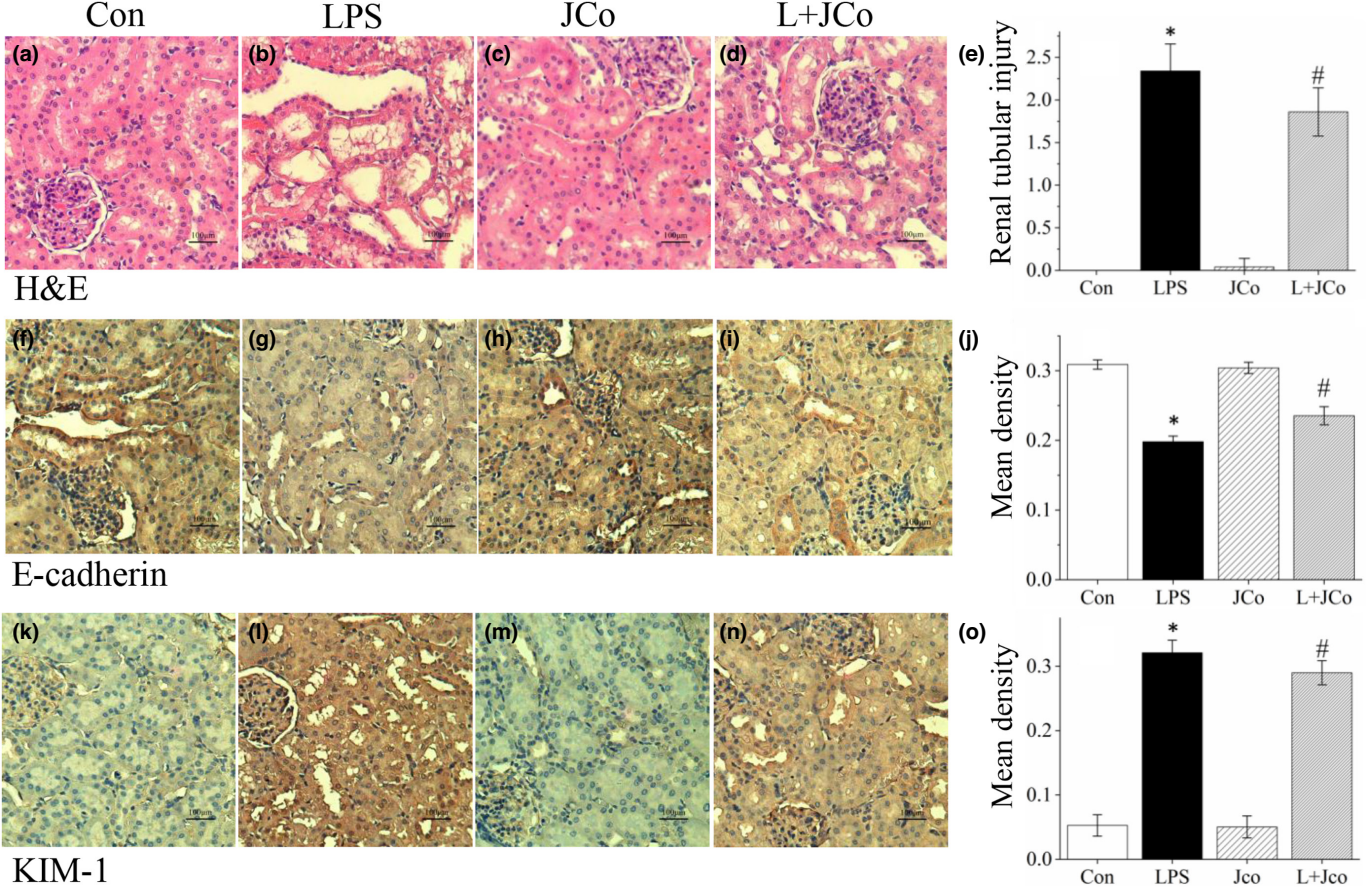
Treatment with JCo extract induced histopathologic changes in the kidney after endotoxic shock in rats. H&E staining (200× magnification) results: (a) Control group, (b) LPS group, (c) JCo group, and (d) LPS + JCo group. (f–i) Immunohistochemical staining of E‐cadherin. (k–n) Immunohistochemical staining of KIM‐1. (e) Semiquantitative analysis of renal tissue injury score in rats. (j) Semiquantitative analysis of E‐cadherin‐positive immunohistochemical staining scores. (o) Semiquantitative analysis of KIM‐1‐positive immunohistochemical staining scores. Semiquantitative analysis results of positive immunohistochemical staining scores are presented as mean density and integrated optical density/area (μm^2^). *p < .05 for the LPS group compared with the control group. ^#^ *p* < .05 for the LPS + JCo group compared with the LPS group. L + JCo and LPS + JCo group

Histopathological analysis using hematoxylin and eosin (H&E)‐stained tissue sections from the kidney after LPS induction revealed renal tubular dilatation, brush border loss, tubular cell swelling, and nuclear loss in the kidney (Figure 4A–D). To investigate the protein expression of KIM‐1 and E‐cadherin, immunohistochemical staining (IHC) was performed. After endotoxic shock induction, E‐cadherin expression (Figure 4F–H) significantly decreased and KIM‐1 expression (Figure 4K–N) significantly increased in the LPS group. Following JCo treatment, the LPS + JCo group exhibited lower renal tubular injury scores (*p* < .05; Figure 4E) and KIM‐1 expression (*p* < .05; Figure 4O) and preserved E‐cadherin expression compared with the LPS group (*p* < .05; Figure 4J).

### 3.3 JCo extract attenuated LPS‐induced overexpression of TNF‐α and IL‐1β through the AMPK pathway

**FIGURE 5:**
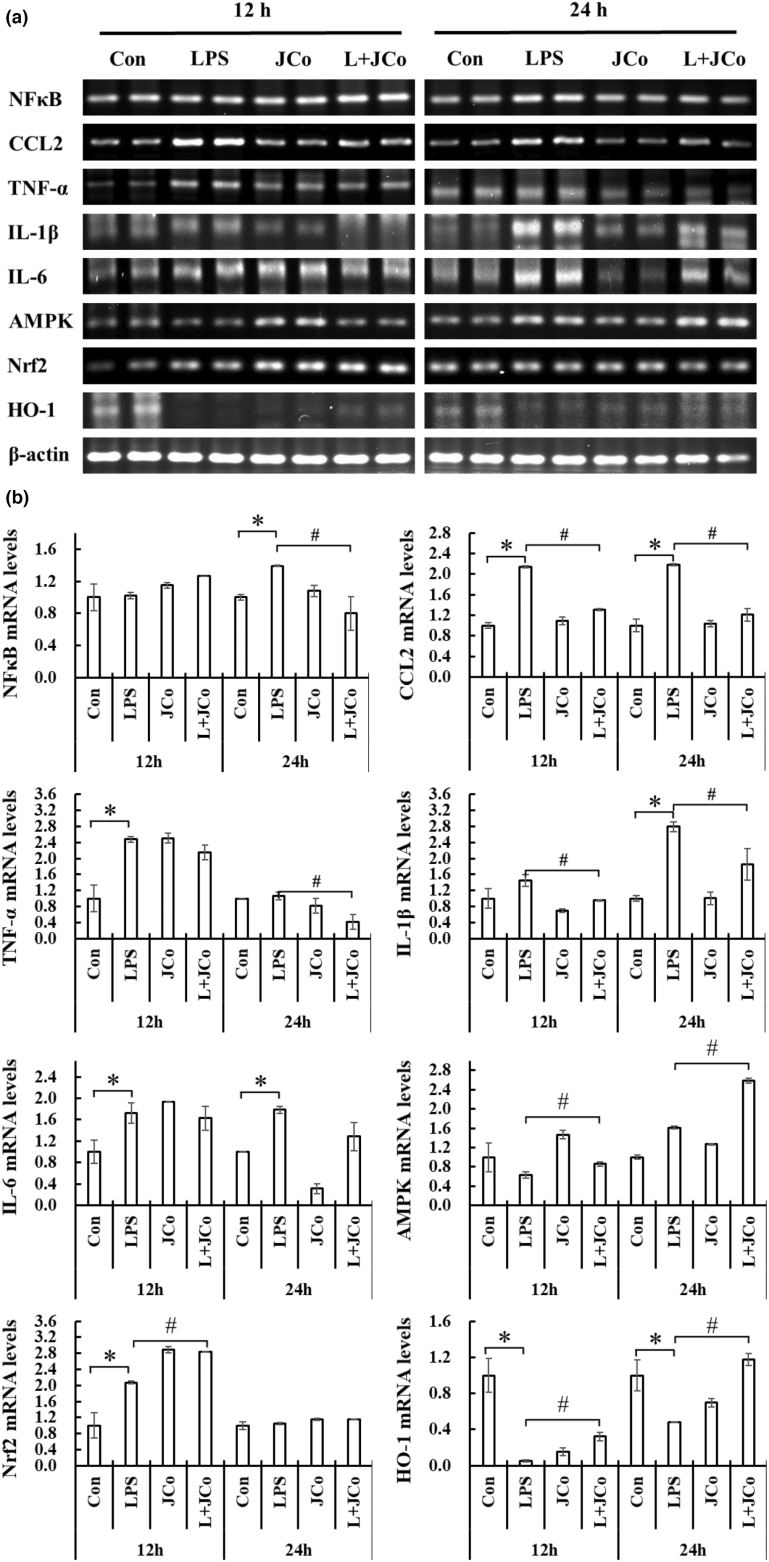
Effect of JCo extract on mRNA levels of LPS‐induced secretion from NRK‐52E cells. (a) Result of RT‐PCR. (b) Results of semiquantitative RT‐PCR analysis of NFκB, CCL2, IL‐1β, IL‐6, AMPK, Nrf‐2, and HO‐1 expression. The mRNA level was calculated as follows: (sample intensity / β‐actin intensity of sample) / (control intensity / β‐actin intensity of control). *p < .05 for the LPS group compared with the control group. ^#^ *p* < .05 for the LPS + JCo group compared with the LPS group. L + JCo and LPS + JCo group

Because LPS‐induced inflammatory response and oxidative stress increased TNF‐α and IL‐1β expression, we investigated whether JCo suppresses their expression in NRK‐52E cells. The results of the semiquantitative reverse transcription PCR (RT‐PCR) experiments after treatment with JCo indicated that the mRNA expression of NF‐κB, monocyte chemoattractant protein‐1, IL‐1β, and IL‐6 (Figure 5A) was suppressed in NRK‐52E cells (Figure 5B).

**FIGURE 6:**
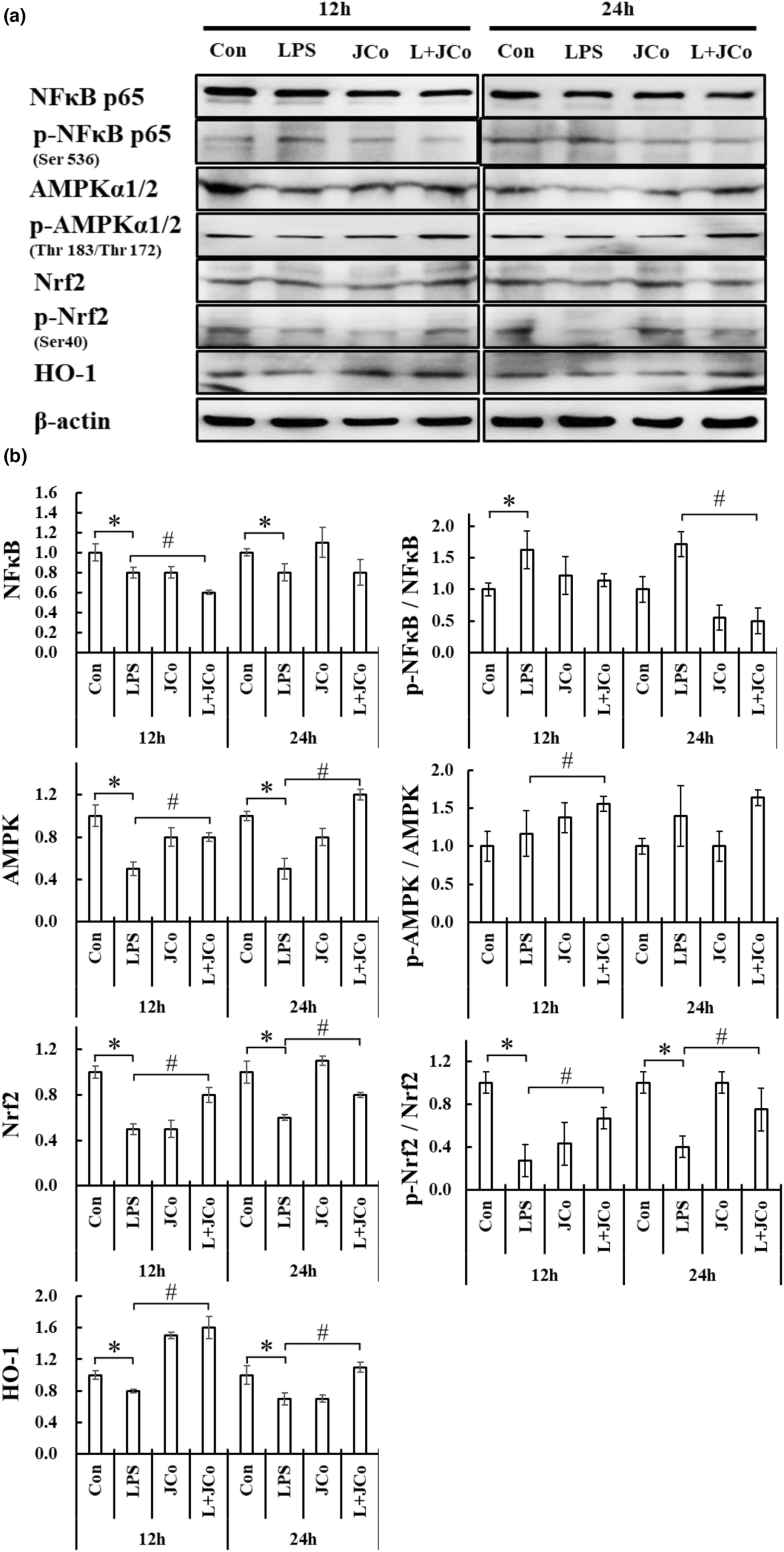
Western blotting of NRK‐52E cells 12 or 24 h after incubation with or without 10 μg/ml LPS or 10 μg/ml JCo extract. The mediators of inflammation were NF‐κB p65 and phospho‐NF‐κB p65 (at serine 536), AMPK and phospho‐AMPK (at threonine 183 and threonine 172, respectively), Nrf2 and phosphor‐Nrf2 (at serine 42), and HO‐1. The band of each sample was quantified using ImageJ 1.47 t software, and all results were normalized to the value of the control β‐actin. *p < .05 for the LPS group compared with the control group. ^#^ *p* < .05 for the LPS + JCo group compared with the LPS group. L + JCo and LPS + JCo group

A Western blotting analysis revealed that JCo extract exerted a similar suppressive effect on the LPS‐induced production of NF‐κB, whereas the levels of AMPK, Nrf2, and HO‐1 (Figure 6A) were significantly restored 12 and 24 h after cotreatment with JCo and LPS (Figure 6B).

### 3.4 JCo extract attenuated oxidative stress in LPS‐induced NRK‐52E cells

**FIGURE 7:**
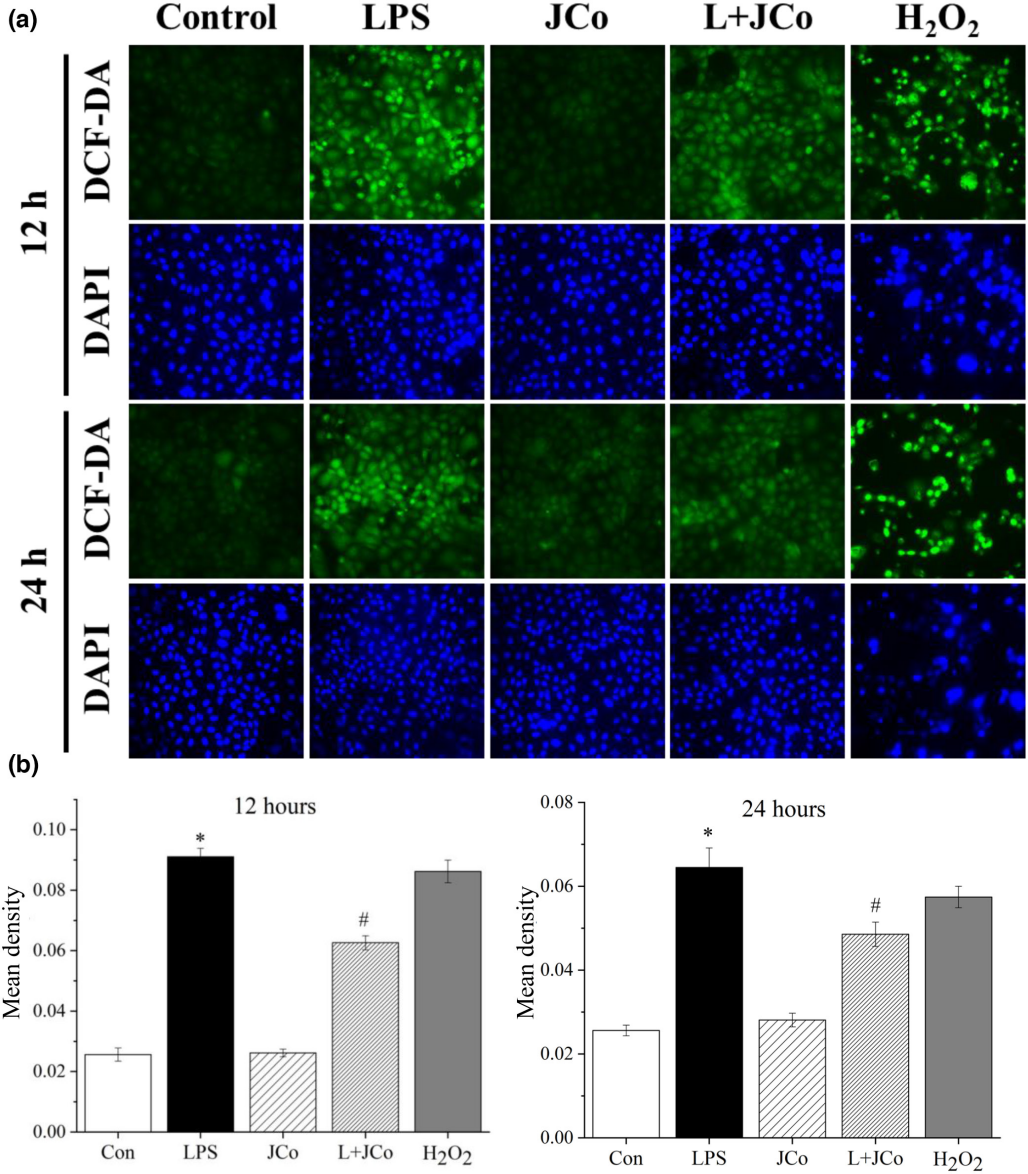
JCo extract reduced LPS‐induced ROS production. (a) ROS assay in NRK‐52E cells 12 and 24 h after LPS treatment. (b) Semiquantitative analysis of ROS expression by using image‐pro plus 6.0. *p < .05 for the LPS group compared with the control group. *p < .05 for the LPS + JCo group compared with the LPS group. L + JCo and LPS + JCo group

We examined the antioxidant activity of JCo extract in the rat kidney cell line. In the ROS assay, an increased fluorescence intensity of DCF‐DA was observed after LPS treatment (Figure 7A). JCo extract significantly reduced DCF‐DA fluorescence intensity in NRK‐52E cells 12 h after LPS treatment (Figure 7B).

## 4 DISCUSSION

**FIGURE 8:**
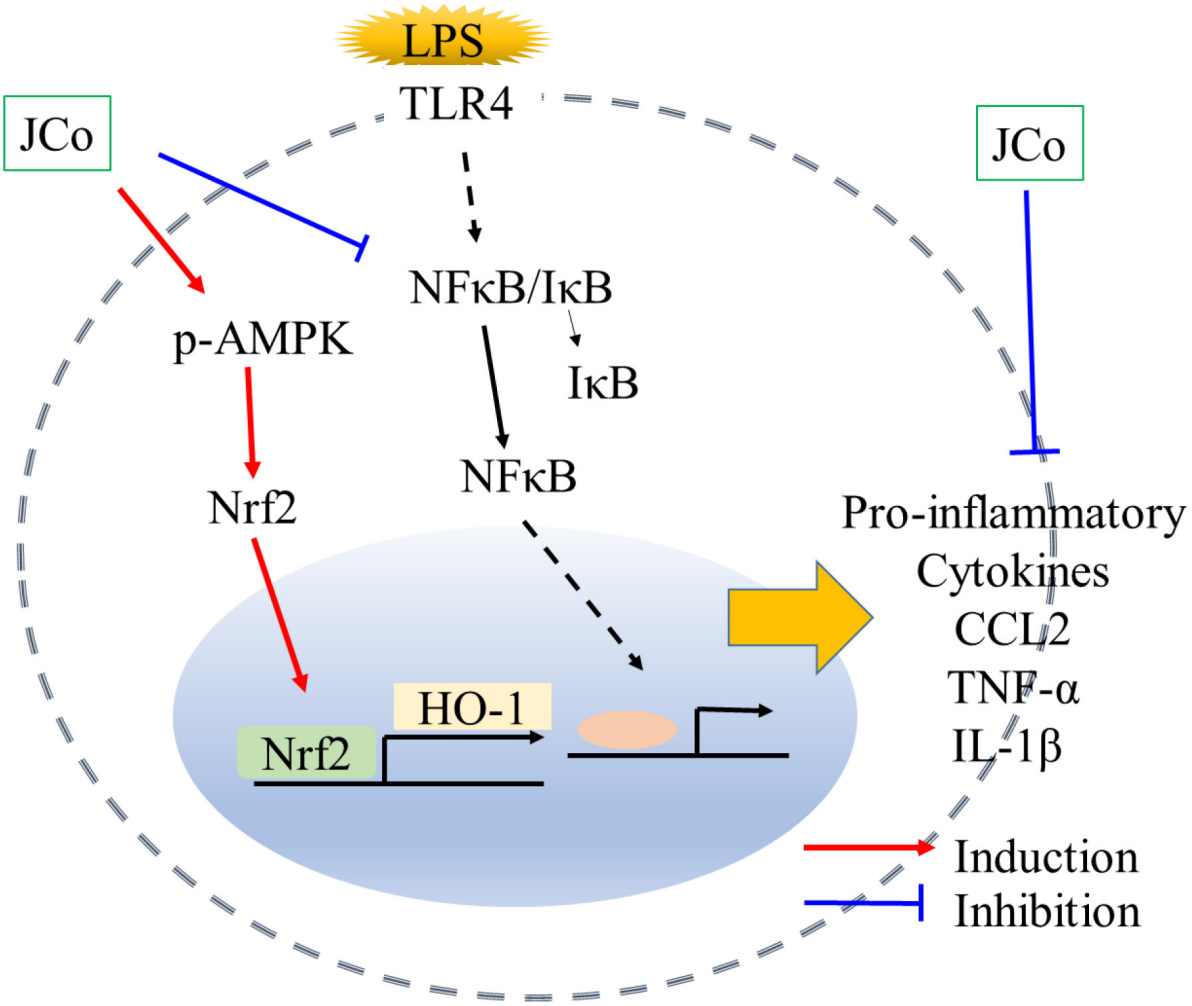
Hypothetical mechanism of the protective effect of JCo extract against LPS. LPS exposure leads to NFκB‐mediated inflammatory response, which increases TNF‐α and IL‐1β expression. JCo extract may reduce inflammatory mediators and oxidative stress through the activation of the AMPK pathway

We demonstrated the involvement of the AMPK‐mediated pathway with JCo extract treatment in LPS‐induced inflammation and oxidative stress (Figure 8). LPS‐induced inflammatory response is a well‐established model for studying inflammation‐induced organ damage (Doursout et al., 2016). Our data indicated that LPS induced a significant reduction in arterial blood pressure and increase in proinflammatory cytokines such as TNF‐α and IL‐lβ. Our data also revealed that JCo treatment did not reduce LPS‐induced hypotension, suggesting that the administration of JCo extract was insufficient to maintain physiological homeostasis in our rat model.

Histopathological analysis of AKI in proximal tubules revealed tubular luminal dilatation, loss of epithelial cell nuclei in some cells, and loss of the brush border (Gaut & Liapis, 2021). An examination of histologic features revealed tubule dilatation, dilatation, brush border loss, and cell sloughing in the kidney 48 h after LPS induction. The kidney injury score of the JCo group was significantly lower than that of the LPS group (*p* < .05; Figure 4E). KIM‐1 is a marker for AKI and is overexpressed in injured renal tubules (Gaut & Liapis, 2021). E‐cadherin acts as a survival factor, and the loss of E‐cadherin is associated with ROS upregulation in invasive ductal carcinomas (Padmanaban et al., 2019). IHC staining on rat kidney tissues revealed that JCo extract reduced KIM‐1 overexpression and restored E‐cadherin levels. JCo extract reduced the serum BUN and Cre levels and ameliorated the histopathological changes that occur in the kidney after endotoxic shock. JCo extract also lowered serum TNF‐α and IL‐1β production after endotoxic shock, possibly owing to its anti‐inflammatory properties.

Lipopolysaccharide activates the PI3K–Akt pathway and induces p65 phosphorylation, leading to increased NFκB activity and NFκB‐mediated gene transcription (Liu & Malik, 2006). NFκB activation induces the expression of cytokines, such as TNF‐α, IL‐1β, and IL‐6, and chemokines, such as CCL2 (Liu et al., 2017). In one study, a water JCo extract inactivated the PI3K–Akt pathway in cancer cells (Raasmaja et al., 2019), and our data revealed that JCo extract suppressed NFκB expression and the downstream expression of chemokines and cytokines such as TNF‐α, IL‐1β, IL‐6, and CCL2 in the rat renal cell line NRK‐52E. In addition to the inflammatory response, LPS induced IL‐1β mRNA expression, controlled by nod‐like receptor protein 3 inflammasome, which is involved in the AMPK–ROS signaling pathway (Wang et al., 2018). Our data revealed that JCo extract increased AMPK and HO‐1 expression and attenuated ROS overproduction, which led to a reduction in renal tubule damage and a higher survival rate in our rat model. The results revealed that JCo extract ameliorated LPS‐induced AKI by attenuating NFκB and ROS overproduction and restoring the AMPK pathway. These findings may provide insight into the use of natural products to manage and prevent bacterial infection and sepsis.

## CONFLICT OF INTEREST

The authors have no conflicts of interest to declare.

## ETHICAL APPROVAL

The study protocol was reviewed and approved by the Institutional Animal Care and Use Committee of Tzu Chi University of Science and Technology, approval number 2017004.

## Supporting information


Table S1

## Data Availability

All data generated or analyzed during this study are included in this article. Further enquiries can be directed to the corresponding author.
