# Supplementary material for: Juniperus communis extract ameliorates lipopolysaccharide‐induced acute kidney injury through the adenosine monophosphate–activated protein kinase pathway
Source: Food Sci Nutr. 2022 Jul 2;10(10):3405–14. doi: 10.1002/fsn3.2941 (PMC9548363; doi:10.1002/fsn3.2941)
Supplement: Supplementary file 1 — Table S1 [file FSN3-10-3405-s001.docx]

Supplement

Table 1. Primer sequences used for semi-quantitative Reverse Transcription-Polymerase Chain Reaction analysis.

|  | Forward Primer (5’-3’) | Reverse Primer (5’-3’) |
| --- | --- | --- |
| NF-κB | AACAACACAGACCCAGGAGT | CTGTCACCAGGCGAGTTATAG |
| CCL2 | GTCACCAAGCTCAAGAGAGAGA | GAGTGGATGCATTAGCTTCAGA |
| TNF-α | CGAGTCTGGGCAGGTCTACTTT | AGAGGTTGAGGGTGTCTGAAGG |
| IL-1β | CCCTGAACTCAACTGTGAAATAGCA | CCCAAGTCAAGGGCTTGGAA |
| IL-6 | GAGTTGTGCAATGGCAATTC | ACTCCAGAAGACCAGAGCAG |
| AMPK | CGGCAAAGTGAAGGTTGGCAAA | CAAATAGCTCTCCTCCTGAGAC |
| Nrf2 | TCTGACTCCGGCATTTCACT | GGCACTGTCTAGCTCTTCCA |
| HO-1 | CACGCATATACCCGCTACCT | CCAGAGTGTTCATTCGAGA |
| β-actin | AAGTCCCTCACCCTCCCAAAAG | AAGCAATGCTGTCACCTTCCC |
